# Factors Affecting Users’ Satisfaction with Urban Parks through Online Comments Data: Evidence from Shenzhen, China

**DOI:** 10.3390/ijerph18010253

**Published:** 2020-12-31

**Authors:** Ruixue Liu, Jing Xiao

**Affiliations:** School of Architecture and Urban Planning, Shenzhen University, Shenzhen 518060, China; lrxrainbow@szu.edu.cn

**Keywords:** urban parks, users’ satisfaction, online comment data, textual analysis, sentiment analysis, Dianping

## Abstract

It is essential to give full consideration to the potential barriers facing urban parks from their better functions and meeting residents’ needs in terms of collective perception and satisfaction. This paper presents the methods of using social media (Dianping) data to investigate the potential factors affecting people’s satisfaction with urban parks in Shenzhen, China. Textual analysis and sentiment analysis make it feasible to identify the factors influencing people’s experience in parks. By measuring emotions towards these factors, a multiple linear regression model helps to explore the relationships between the factors and people’s satisfaction, and among them, determines the key ones. The results present the nine key factors of urban parks that affect the users’ satisfaction, in addition to the common factors by previous studies including park size, vegetation, recreation facility, landscape visual effect, maintenance of facilities and plants, and environment cleanliness. A series of contextual factors also significantly influence people’s satisfaction, such as sign system, mosquito and air quality. Among these, sign system has the strongest influence. The results increase the understanding of the human-urban park relationship and identify the characteristics of urban parks that facilitate the degree of satisfaction promotion. Our findings may provide the managerial guidelines for planners and decision-makers to optimize people’s imperative qualities of urban life.

## 1. Introduction

Urban areas confront issues of increasing population concentration in the last decades. Agglomerations that have over 300,000 people accounted for 57% of the entire population by 2014, and the reports estimate that, by the year 2030, 62% of the global population will live in urban areas [1]. To provide a positive urban environment that contributes to the quality of life becomes a major challenge. As cities expand and grow denser, urban green spaces, particularly parks, are gaining an increasing amount of attention from both academic and policy arenas. The potential benefits that parks could offer to urban residents are increasingly significant in an urbanized society, given their potential to improve environmental qualities, human health and neighborhood livability [2]. A growing body of literature highlights many ecological, socio-cultural and economic benefits provided by urban parks [3,4,5]. Therefore, better understandings of the interactions between urban population and parks can provide managerial guidelines for planners and decision-makers to optimize people’s imperative life qualities. Such guidelines can be useful for local authorities to plan and manage urban parks more effectively, and promote a higher level of incorporation of functional and usable urban parks into urban life.

Decision-makers and urban planners are inclined to the idea of the livable urban environment [6]. However, livability strongly depends on people’s expectations on an individual scale. To understand people’s perceptions and satisfaction with urban parks is, therefore, fundamental to improve urban livable and societal well-being [7]. Knowing people’s perception and whether they are satisfied with urban parks is important, as well as an awareness of the potential barriers that may impact people’s satisfaction and consequently prevent parks from being under proper use [8]. Individual perception and satisfaction are also important for successful urban design and management of urban parks because they influence choices of destination, utilization of parks, and decision to return [9]. Information on perceptions, satisfaction, and attitudes of people may serve as an important factor for the policymaking, planning, and management of urban parks [10]. Therefore, for a better meeting for urban residents’ needs and making full use of parks, the preliminary phase of planning urban parks has to take full consideration of the opinions of users in general [11]. It will assist urban managers to provide better services and consequently contributing to people’s quality of life [7].

To identify the relationship between human and urban parks has recently become a subject of scientific interest. A considerable amount of research investigates people’s utilization and satisfaction with urban parks, as well as influencing factors. For example, physical attributes of parks have been found to influence people’s experience, including the accessibility to and size of parks, as well as the quality of vegetation [12,13,14]. Availability and status of public facilities are also closely correlated with people’s experience. It addresses the cases like benches for the elderly, playgrounds for parents with small children, places of walking dogs off-leash for dog owners, and bicycle paths for cyclists [15,16,17]. The mere presence of facilities is, however, insufficient without proper maintenance [16,18]. The absence or poor quality of management and maintenance elicits people’s negative perceptions. For example, people may have a negative recreational experience when seeing litters at the beginning of trail; they often evoke fear of crime when observing signs of vandalism and become allergic when finding plants with high allergenic potentials [19,20,21,22,23]. Physical attributes, facilities, management and maintenance of parks are internal factors with a direct influence on people’s perceptions and satisfaction. Meanwhile, several external factors not directly connected with people’s perception and satisfaction in parks has potential influences on people’s experience. First, users’ behavior is one of the key factors of this kind. Misbehaviors of other users lead to common negative impressions. Further examples include that people prefer quieter places to avoid crowds, and those caring youngsters would dislike dogs off-leash [24,25]. Second, socioeconomic factors of park users such as age, gender, education, occupation, and income are also relevant to people’s feeling within parks [17,26,27]. Besides, there are additional variables that equally affect individual opinions, such as the frequency of organized activities [28,29].

Previous attempts to examine people’s perception and the degree of their satisfaction with urban parks have followed traditional methodological approaches. Many rely on qualitative, report-based approaches in which surveys and interviews provide information from participants on a range of topics [7,10]. Self-reporting techniques also help to examine how and when human populations using urban parks [30,31]. Subjective report-based methods, as such, may have limitations for further extensive application. For example, total reliance on participants’ responses may lead to social biases in regards to desirability, and it is difficult to validate the information received from participants. These methods are also usually site-specific and time-consuming and include only small groups of participants, as well as other limitations such as participant recall bias [32]. Indeed, validations of the questionnaire responses reveal inconsistency [33]. Consequently, if only using traditional methods of subjective reporting, it imposes challenges for the measurement of human interactions with urban parks in a more engaging manner. 

It echoes the fact that new technologies and especially advanced social networks have changed urban lives intensely. One of the changes is that people connected via the Internet can easily post their comments and experience of their visits on websites [34,35]. The emergence of free social media data available online may put forward advance assessment techniques of human interaction with urban parks—that is, how, when and why people use them, which activities occur within them, and how people feel while using them. Social networks and social media platforms enable people connected to the Internet to provide information about their locations, feelings and activities [36,37]. As such, they provide a source of sensing and information to understand the motivational factors hidden behind the perplexing habits of a large population [38,39]. Information from social media and big-data sources increases recently, and it demonstrates how people interact with real environments and make it possible to evaluate user perceptions and satisfaction with spaces. In other words, besides conventional approaches, new data and methods may be useful for studying urban parks, driving forces of their use and nonuse, as well as people’s perception and satisfaction with them. 

While the application of social media data in urban park research is still in its infancy, a growing number of studies have used it in the fields of geography, landscape and urban planning, tourism management and urban ecological environmental protection over the past few years [40,41,42,43,44,45,46]. It claims that social media data with nearly real-time monitoring do assist multiple scientific types of research and practices. Accordingly, in urban parks research, the multiplicity of digital traces that people leave on social media platforms—from text to images, tags and shared locations—can provide new insights about the popularity, function and perception of urban public spaces [47,48]. The approach benefits researchers extensively by reducing both time and financial costs, given that free data are available to download at a time interval as frequently as necessary. Access to social network data may also provide a larger sampling coverage with a range of intra-urban or even global zones, as well as for any period ranging from a few hours to several years [28]. In this sense, traditional surveys and interviews are often impotent when facing with heterogeneous data and diversified users. 

Data from web-shared services may largely contribute to monitoring human interaction with urban parks. Recently, a number of studies have used the increasingly available social media data, like that on Twitter, Instagram or Flickr to measure people’s experience within urban space, especially in developed western countries [41,49,50]. However, few studies use data to estimate human interaction with urban parks in developing countries with larger populations like China, without considering the issues of urban public space concerning people’s perception and satisfaction [34,51]. While understanding people’s experience within parks is important for planning and managing high-speed urbanization, research on people’s perceptions of and satisfaction with urban parks is extremely limited in Chinese cities due to the lack of reliable data on people’s feelings. Consequently, people’s experience and needs receive little attention during park design, construction, and management processes. With more available social media data, we expect to use them as a potential proxy for both quantitative and qualitative studies on individual perceptions toward urban parks.

Our study takes online comment data from the online platform Dianping as typical social media data. We use them as a proxy of people’s experiences to derive people’s perception of and satisfaction with urban parks. In this exploratory study, we study Dianping as a means of commenting on a topic. We expect to identify the experience of park users and the key factors that affect their satisfaction. The study was conducted in Shenzhen, one of the most important metropolitan cities and the fastest growing cities in China. While achieving economic development miracles and rapid urbanization, Shenzhen increasingly confronts land shortage, population pressures and environmental problems, all of which hamper the sustainable development of the municipality [52,53]. A growing imbalance between the supply and demand of open urban spaces is discernible [54,55]. Therefore, the government has made efforts to optimize urban living environments by constructing and upgrading the quality of urban parks. Specifically, the objectives of this study are: (1) to assign the potential factors affecting people’s perception of and satisfaction with urban parks based on the online comments data from Dianping; (2) to explore the relationship between these factors and people’s satisfaction, and further identify the significant factors. The results of this study can optimize urban park planning and management according to users’ expected perceptions.

## 2. Materials and Methods 

### 2.1. Study Area

The territory of Shenzhen locates between longitudes 113°45′44″ E and 114°37′21″ E and latitudes 22°26′59″ N and 22°51′49″ N. Before China’s economic reform and the open-door policy in 1978, Shenzhen’s economic development was quite slow for years. Its population in 1979 was only 314,100 with the gross domestic product (GDP) of 196 million RMB [52]. Since the establishment of the Shenzhen Special Economic Zone in 1980, Shenzhen has developed into a modern metropolis with well-developed secondary and tertiary industries. According to the Shenzhen Municipal Bureau of Statistics, by the end of 2019, the city had ten administrative regions. The total administrative division area was 1997.47 km^2^, and the permanent population was 13.439 million, with a gross domestic product of 2693 billion RMB [56]. 

**Figure 1:**
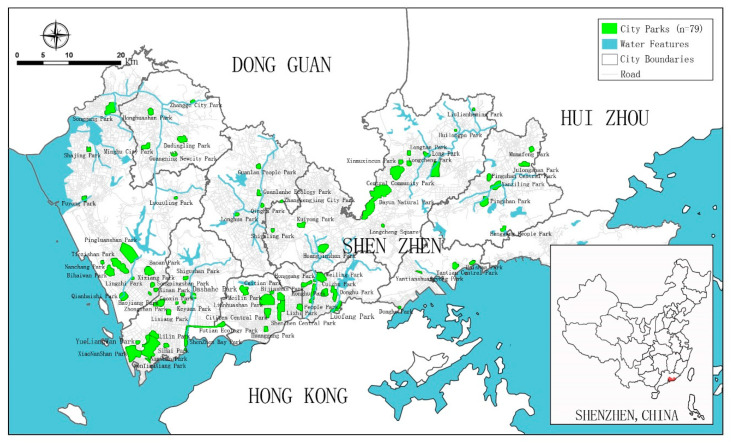
The distribution of sample parks (drawn by the authors).

Considering the statistics on urban parks provided by the Shenzhen Municipal Bureau of Statistics at the end of 2019, a total of 1090 parks under registration had a total area of 39,319 ha [56]. Based on the main intended functions of parks and their official Standard for Classification of Urban Green Space (CJJ/T85-2017), these parks fall into seven categories, which are: comprehensive parks, community parks, theme parks, forest parks, country parks, geological parks, and wetland parks [57]. Comprehensive parks that are suitable for our study may fulfil multiple standards, including: (1) fully equipped with recreation facilities and developed infrastructure; (2) used for activities such as sightseeing, resting, recreation, sports, and research; and (3) provided multiple services for visitors free of charge. Based on this, this study selects 79 comprehensive parks distributed in the region, as shown in Figure 1.

### 2.2. Data Collection and Extraction

This study uses online comments data to analyze the factors affecting people’s general perception of and actual satisfaction with urban parks. The online comment data obtained from the Dianping website (http://www.dianping.com) provide a review platform for business and entertainment. Created and launched in 2003, Dianping is the leading social media platform in China and one of the earliest independent third-party consumer review websites in the world. In the Dianping website, a user would freely comment on the places they have visited without a word limit. Anyone connected to the Internet via smart devices or computers could access the platform and receive and share information. According to Dianping’s annual report, it had 127.729 million monthly active users in December 2017, accounting for more than 9% of the Chinese population. It makes Dianping a highly influential player in the distribution of information and opinions. As a typical open crowdsourcing data, Dianping data are the most appropriate dataset that one could obtain as a proxy to estimate people’s perception of and satisfaction with urban parks. Information obtained from Dianping has already been used successfully in a diverse array of urban lives in China, including commerce trend, catering enterprise brand image, personal consumption behavior, and tourists’ preference of scenic areas [58,59]. While Dianping data has proved to be useful to investigate people’s urban lives in general [60], it is not clear to use it for the study of public urban spaces, especially urban parks.

**Figure 2:**
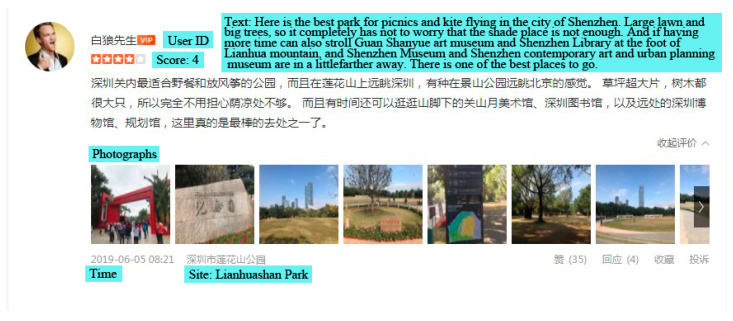
A screesnshot of online comments from the Dianping website (From http://www.dianping.com/shop/H6Z7zyY6lwRs0nTN/review_all). The English translation of Chinese characters in the figure is presented in the blue box.

To create a corpus of online comment data for investigation, Chinese language comments were downloaded via Dianping application program interface (API) using the names of 79 comprehensive parks as queries, for instance, ‘Lianhuashan Park’ and ‘Shenzhen Bay Park’. It is the only search term to obtain the full range of comments related to each comprehensive park. This methodology creates a corpus of data that contain comments on the comprehensive parks in Shenzhen. The comments capture a range of their information, including dates of comments, general evaluating scores, texts, and photographs taken within. A sample of comments from the Dianping website is presented in Figure 2 and Table 1, demonstrating a variety of information that comments contain. Scores range from 1 to 5, with 1 standing for dissatisfied, 2 neutral, 3 good, 4 satisfied, and 5 very satisfied. These scores are helpful to quantify the people’s satisfaction with selected parks.

The comment data obtained from Dianping are then manually screened to ensure that they are relevant to the specified urban park. Duplicates and those comments that are irrelevant to the topic or for commercial purposes are removed. Comment data have a coverage of nine years from June 2011 to June 2020. The processed data return a total of 11,272 comments on the selected comprehensive parks.

### 2.3. Analysis

#### 2.3.1. Textual Analysis

Textual analysis is a qualitative and exploratory procedure to identify and determine the potential factors influencing people’s experience in parks by grouping them into topic nodes. The nature of comment texts is qualitative. There are two approaches for qualitative data analysis: inductive and deductive. For example, the analytic method for representing inductive is a grounded theory, which is a “way of arriving at the theory suited to its supposed uses” in contrast to the theories “generated by logical deductive from a priori assumptions” [61]. In the deductive analysis, the examination of qualitative data, such as interview transcripts or users’ comments, has a feasible framework [62]. As there is no established framework to select factors, the inductive approach seemed more appropriate for this study. We aim to capture important themes about actual visitors’ experience within parks by the inductive approach. The potential discovery of those key factors, which influence such experience, may help to understand the reasons behind the perceived strengths and weaknesses of planning and management of urban parks. 

The coding process is an important analytical strategy in inductive qualitative analysis. The analysis involves a two-step procedure: first, the analyst conducts “open-coding”, where texts are broken down into components and refined into abstract groups; second, the analyst conducts “selective coding”, which entails a selection of major cores, most frequently used significant categories or variables to synthesize data [63,64]. Coding means “applying a short-hand label to sort, synthesize, and conceptualize data”. The process of coding begins with the identification of keywords or codes. Codes refer to “the word or short phrase that symbolically assigns a summative salient, essence-capturing, and/or evocative attribute for a portion of language-based or visual data” [65]. Through open-coding, each of the qualitative data in question will have a special label; for instance, a sentence in the comment on the accessibility to a park in terms of convenient transportation may have a code of “accessibility”. Every sentence may have a code, so that one comment text may contain several codes simultaneously.

#### 2.3.2. Sentiment Analysis

Sentiment analysis targets individual opinions, attitudes and emotions towards entities such as products, services, organizations, locations and events [50,66]. Comment texts can be used to measure visitors’ emotions, an important concept in relation to their attitudes. Sentiment analysis may help to measure sentiments in comment texts. Sentiment scores of every factor in individual comment text are assigned by a manual annotator into one of three categories: positive, neutral, or negative. This annotation relies on the presence of emotive words, emotions or meanings in comments [67]. If any, images uploaded alongside comment texts may aid annotation and provide the annotator with more contexts. The chosen high-level emotions include Ekman’s six basic emotions (anger, disgust, fear, sadness, happiness, and surprise) as well as love and calmness, in line with previous researches that undertook sentiment analysis of Twitter data [68,69]. These emotions fall into two categories: 1. positive, including calmness, happiness, love, and surprise; 2. negative, including anger, disgust, fear, and sadness. Beauty stands for a subcategory of positive texts, but outside of emotions, to account for a large number of texts referencing the beauty of landscape. Based on the strongest presented emotions, each factor within texts could only remark on one of these emotion categories. All factors cannot be mentioned in one-comment texts. Several factors are mentioned, while others are not mentioned in most comment texts. Meanwhile, some factors mentioned in comment texts are not presented in any emotive words or consideration. Therefore, if the sentence expresses emotionally as positive, the sentiment score of the factor is equal to [1], emotionally negative [−1], and emotionally neutral or fail to mention [0].

One researcher annotates all of the Dianping data in the sample. The other three researchers annotate 1000 random comments to assess the reliability of different human annotators. A metric of comparison is derived (K = 0.736), suggesting sufficient inter-annotator reliability [70].

#### 2.3.3. Statistical Analysis

A multiple linear regression model helps to explore the relationships between potential influential factors and people’s satisfaction with parks. This model estimates the response variable (satisfaction) and is estimated by using the mean value of the score on a general evaluation of each park and the predictor variables (influential factors) are determined by sentiment analysis on online comments. Using the variables above, the multiple linear regression model appears as follows:

Y = b + a_1_X_1_ + a_2_X_2_ + a_3_X_3_ + …… a_n_X_n_

With the aim of comparing each potential factor’s influence the response variable, and the study normalizes all predictor variables before the regression.

This research develops a series of potential influential factors to identify the level of satisfaction of each determinant, while taking overall satisfaction as the response variable. We calculate the variance inflation factor (VIF) for each predictor variable and the mean VIF to test the multicollinearity among predictor variables, as some of them are correlated. We conduct all statistical analyses using SPSS 22.0. 

## 3. Results

### 3.1. Characteristics of People’s Online Comment on Parks

**Figure 3:**
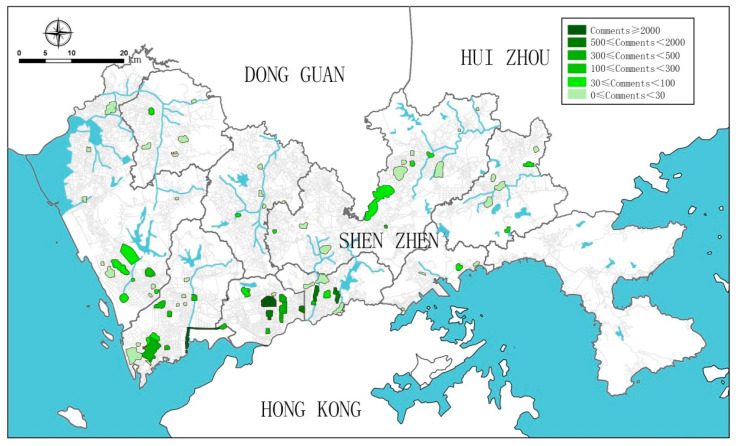
Distribution of comments to Shenzhen’s comprehensive parks measured by Dianping data (Drawn by the authors).

This study collected a total of 11,272 comments relating to the 79 case-study sites over nine years of data capture, with an average of 143 comments per park, a standard deviation of 393, and a range from 0 to 2511. The distribution of social media data is highly skewed. The majority of these parks (n = 61) have less than 100 comments, and 13 parks have no comment. Nine parks have more than 300 comments and six parks (7.6% of the total) have more than 500 comments. These six parks have a total number of 7344 comments, accounting for 65.57% of the total (Donghu Park, Honghu Park, Lizhi Park, Lianhuashan Park, Civic Center Park, and Shenzhen Bay Park). The number of comments varies greatly by location (Figure 3). The number of comments is greater in southwest areas, especially for the large parks such as Lianhuashan Park and Shenzhen Bay Park. The parks in northern and eastern areas receive far fewer comments.

**Figure 4:**
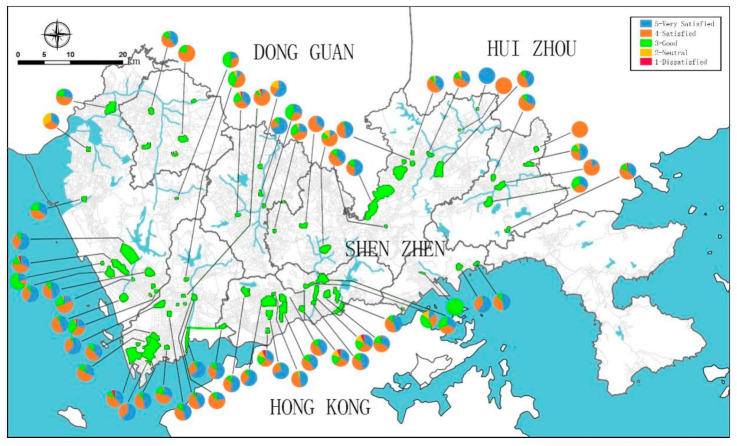
The proportion of dissatisfied, neutral, good, satisfied, and very satisfied users’ satisfactions received per comprehensive park (drawn by the authors).

Excluding 13 parks without comments, the mean score of people’s satisfactions with comprehensive parks in Shenzhen is 4.16, falling between the categories of “satisfied” and “very satisfied”. The majority (64.56%) of the mean scores of users’ satisfaction with each park falling between 4 and 5, but 15 parks in general evaluation have the mean score between 3 and 4 (Table 2). The proportions of the scores of users’ satisfaction received from the discrete comprehensive parks are highly variable in terms of spatial variation (Figure 4). For example, the vast majority of the emotional responses captured in Lanhuashan Park, Shenzhen Bay Park, Zhongshan Park, and Civic Center Park are very satisfied. The majority of emotional responses in Cuizhu Park, Bijiashan Park, and Dashahe Park are satisfied. In contrast, more than 30% of the emotional responses captured in Xinan Park, Shiyaling Park and Longhua Park are good, neutral or dissatisfied. It proves applicable to identify people’s satisfaction with urban parks using Dianping data, which represents a useful resource for urban planners and park management.

### 3.2. Factors Influencing People’s Experiences within Urban Parks

The first round of coding identifies a total number of 39 codes that are relevant to the factors of people’s experience in parks. Many of the identified factors, such as traffic to reach, park size, plant, and facility are popular in previous studies. There are also new factors in the coding process, such as mosquito, patrolling security service, and direct the flow of people. The second iteration revisits these codes with the aim of consolidation. For instance, the codes of “play equipment” and “sports field” attach to “recreational facilities”, and “litter bins” and “toilet” more with “sanitation facility”. This step results in the categorization of the 39 codes into 21 higher-level codes. The final round further consolidates the remaining codes into six categories. These categories represent the main potential aspects influencing people’s feelings.

**Figure 5:**
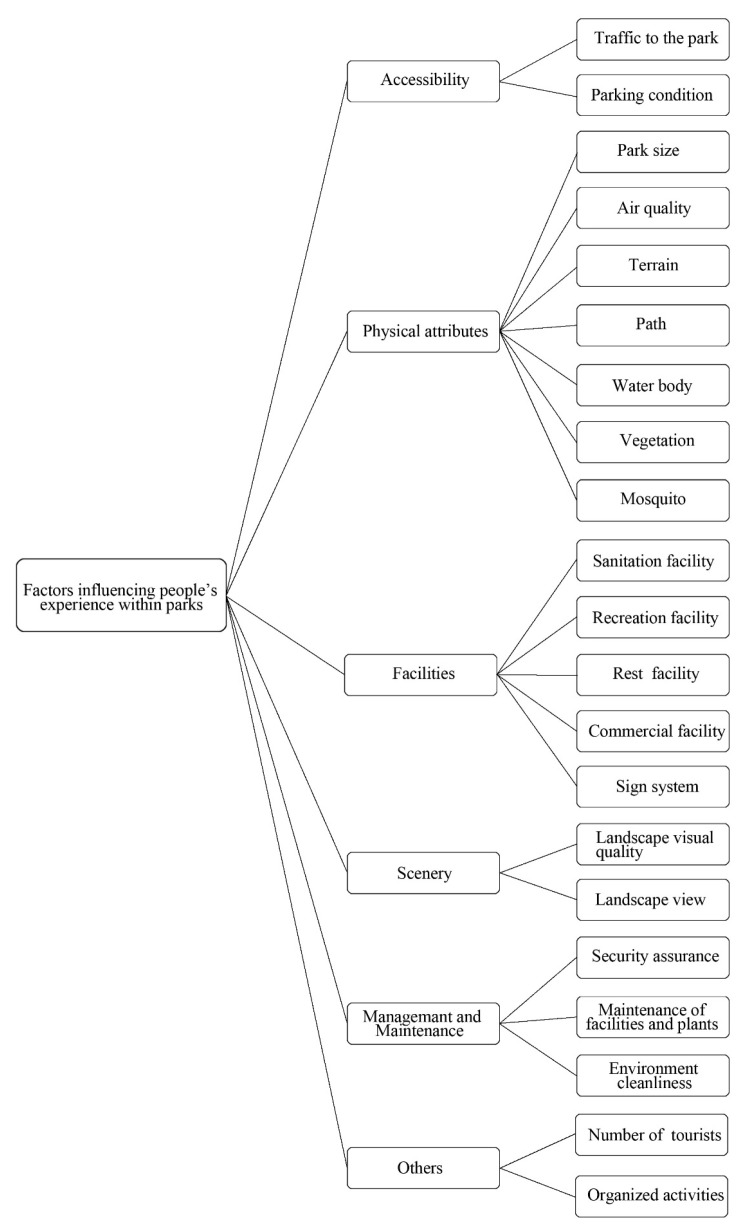
Factors influencing people’s experience within the parks (drawn by the authors).

As noted previously, the inductive process results in six major factor categories. They are accessibility, physical attributes, facilities, scenery, management and maintenance and other factors (Figure 5). It is apparent that, in addition to the core factors such as accessibility, physical attributes facilities and scenery, contextual factors such as management or maintenance of parks and other factors such as the number of tourists and organized activities appear to influence people’s feelings within urban parks.

**Figure 6:**
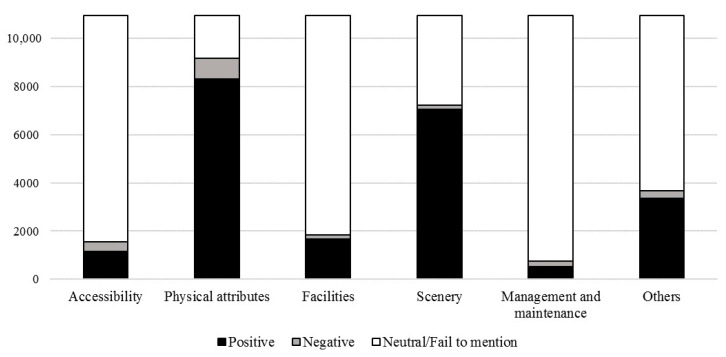
The number of online comments assigned to positive, negative and neutral emotions/fail to mention each factor category.

**Figure 7:**
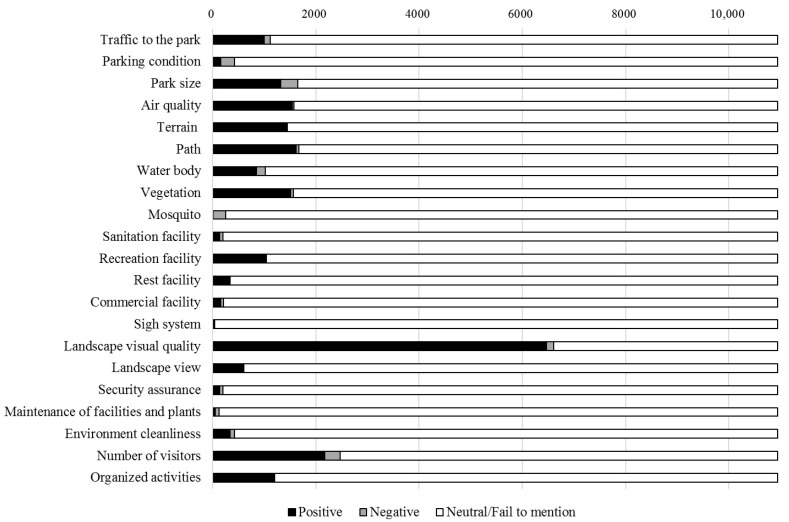
The number of online comments assigned to positive, negative and neutral emotions/fail to mention each factor.

Physical attributes of parks show prominent features among all visitors who had left comments (Figure 6). 83.93% of the total online comments are assigned to positive or negative emotions about the physical attributes of urban parks. Among the seven factors of physical attributes, path had the highest frequencies of positive or negative emotion, followed by park area, air quality, vegetation, terrain, water body and mosquito (Figure 7). It indicates that people focus more on paths among physical attributes of urban parks in Shenzhen, and it meets with the individual preferences of stroll and jogging. A typical comment about the path of urban parks is “suitable for jogging and walking”. 

Scenery is the intuitive factor that is most critical in people’s choice. 65.91% of the total online comments are assigned to positive or negative emotion about scenery (Figure 6). Among the factors in the scenery of parks, people pay more attention to the landscape visual quality. 59.08% of the total comments are assigned to positive emotion about the landscape visual quality, and 1.33% of comments are assigned to negative emotion (Figure 7). Besides, 5.50% of the total comments are assigned to positive or negative emotion about the landscape view (Figure 7). Typical comments about the landscape sight are like “climb to the top to overlook the beautiful city scenery in the distance” and “there are too many trees around the top of the hill, blocking the view”. It indicates that people are concerned with not only the landscape inside but also outside the park.

Satisfaction with urban parks depends on the availability of facilities that meets the needs of various user groups. Recreation facility is presented with highest frequencies of positive or negative emotion among five identified factors, followed by rest facility, commercial facility, sanitation facility, and sign system. 9.47% of the total comments are assigned to positive emotion about the recreation facility, and 0.1% comments are assigned to negative emotion (Figure 7). Typical recreation facilities in urban parks of Shenzhen include children’s playgrounds for parents with small children, bicycle paths for cyclists, and sports fields for people who love sports. 46 online comments are assigned to positive or negative emotion about the sign system of urban parks in Shenzhen, which is rarely mentioned in previous research. Typical comments about the sign system are like “there’s no sign, and you don’t know where to go when getting to the intersection” and “the signs are not clear, and the attractions are hard to find”. It indicates that people have unpleasant experiences if sign systems are absent or unclear.

Among the two factors of accessibility, people pay more attention to the available traffic compared to the parking condition. 9.19% of the total online comments are assigned to positive emotion about traffic, and 1.01% are assigned to negative emotion. 1.43% of all the comments are assigned to positive emotion about parking condition, and 2.44% are assigned to negative emotion (Figure 7). With online comments examined, positive emotion about accessibility are assigned to a variety of comments, including the reports like “the subway goes straight to the gate of the park, the traffic is so convenient” and “lots of cars are on weekends but there is a special parking lot”; negative emotion is assigned to a variety of comments, including reports like “the transportation is not convenient, the subway station and bus station are far away” and “there are not many parking spaces, late no space”.

Management and maintenance of parks receive less attention among visitors’ comments in our study, while only 6.86% of the total comments are assigned to positive or negative emotion (Figure 6). Environment cleanliness (3.84%) is presented with highest frequencies of positive or negative emotion among the three factors of management and maintenance of urban parks, followed by security assurance (1.86%) and maintenance of facilities and plants (1.17%) (Figure 7). This result may indicate that the management and maintenance of the urban parks in Shenzhen is generally good, so not attracting much public attention. 

Other factors of urban parks like the number of tourists and organized activities frequently appear in online comments. 22.60% of the total online comments are assigned to positive or negative emotion about the number of tourists (Figure 7). The perception of the number of tourists can be very subtle. Online comments showing the positive emotion about the number of tourists is assigned to a variety of comments including reports like “many people are coming to the park to see the flowers, many families come together, including young and old. A very warm and happy scene” and “the park is crowded, someone is jogging, someone is shuttlecock kicking, some people are playing badminton, and some people are square-dancing. There’s always an activity for you”; negative emotion is assigned to a variety of comments including reports like “usually good, weekend especially too many people, crowded people” and “the park is sparsely populated and seemed deserted”. It indicates that the perception of the number of tourists in urban parks might depend on people’s mood at that time. Parks may have a significant role in supporting the physical activities of local populations. Observation of organized activities appears to be responsible for attracting more people, including large numbers of spectators. 10.99% of the total online comments are assigned to positive or negative emotion about the organized activities (Figure 7). Additionally, most of the comments are assigned to positive emotion; only two comments are assigned to negative emotion. Typical organized activities in the urban parks of Shenzhen are that of flower show and light show. Typical comments are like “there’re so many beautiful chrysanthemums and many precious species. Different variable chrysanthemums are here, such as tree chrysanthemum, large vertical chrysanthemum, cliff chrysanthemum, potted chrysanthemum and ground-cover chrysanthemum” and “light shows are on weekend nights. The Peak Square at Lianhuashan Park is the best place to watch light shows. Beautiful light show, brilliant light change. I think it is more beautiful than the Victoria Harbour light show in Hong Kong. Many shutterbugs bring professional equipment to take photos”.

### 3.3. Effects of the Influential Factors on People’s Satisfaction with Parks and Their Relative Importance 

A multiple linear regression model may help to analyze the mechanisms of impact and to explore the factors influencing people’s satisfaction with parks. Table 3 shows the results from the regression model: the people’s satisfaction with parks having the number of comments exceeding 30 regresses against all 21 predictor variables (N = 36). The results include both unstandardized regression coefficients and standardized coefficients. Standardized coefficients (beta coefficients) may contribute to determining the relative importance of predictor variables. The absolute value of the standardized coefficient is larger; the predictor variable is more important. The results show that the R^2^ is much higher than 0.5 for the model; it means that the model is relatively stable. Additionally, the mean VIF is 1.769 for the model, with VIF for most of the predictor variables less than three. It suggests a weak and ignorable multicollinearity among the predictor variables (Table 3).

For the model including the parks whose number of comments exceed 30, the results of regression analysis shows that the nine predictor variables, including four park physical characteristics variables (park size, air quality, vegetation and mosquito), two facilities variables (recreation facility and sign system), one scenery variable (landscape visual quality) and two park management and maintenance variables (maintenance of facilities and plants and environment cleanliness), significantly affect the people’s satisfaction with urban parks in Shenzhen (Table 3). Among these nine factors, the sign system has the strongest influence, followed by the maintenance of facilities and plants, mosquito, environment cleanliness, air quality, vegetation, park size, landscape visual quality and recreation facility. Two accessibility variables (traffic to the park and parking condition) have no significant effect. Other factors such as the number of tourists, organized activities and sanitation facility also have no significant influence on people’s satisfaction. The model explains approximately 85% of the variation in people’s satisfaction with parks.

## 4. Discussion

In this study, we used online comment data from social media to quantify people’s experience of urban parks and further investigate how different factors affect people’s satisfaction. The findings from this study explain people’s perception of urban parks at the city scale. The method presented here could be useful when comparing other cities. This method was proposed, following the identification of some limitations with the former survey and interview-based methodologies employed, and the successful use of online comment data in a range of urban-related research. It is important to evaluate this method and to highlight its utility compared to other former methods, as well as to identify the limitations apparent in using online comments as a data source to inform the relationship between people and urban environment.

### 4.1. Factors Influencing People’s Satisfaction with Parks and Their Relative Importance

This study reveals that several factors of park characteristics are important determinants of people’s satisfaction with urban parks based on online comment data from social media platforms. Our findings indicate that nine factors, including sign system, maintenance of facilities and plants, mosquito, environment cleanliness, air quality, vegetation, park size, landscape visual quality and recreation facility, explain the large variation of people’s satisfaction in the comprehensive parks in Shenzhen.

The sign system has the most significant relationship with people’s satisfaction with urban parks in the regression model. Previous studies about the human-urban park relationship rarely have similar findings. Parks with a perfect sign system have a higher degree of satisfaction than those with unclear sign systems or not at all. It might explain that parks with sign systems of guidance, propagandas and beautifiers can meet people’s needs as far as possible. The sign system is a public information facility with a directive and explanatory function, which can provide directions, locations, roads, regional conditions and other information that helps people get around in public spaces. In the space, there is a lot of visual information that is closely related to people’s daily lives and travel activities, which affects our living quality. Most people rely on these guidelines and instructions when they walk or move about in public spaces. The sign system acts as a guide, whether detailed, accurate or greatly systematic, influences visitors’ travel experience and knowledge of places. In this sense, parks, especially comprehensive parks, which are kernels in urban public spaces, usually have large and multiple areas with different functions. A clear sign system can help people to know more about the place and to guide people. Previous studies on the sign system in public spaces mainly focus on the sign system of public buildings such as libraries, museums and subway stations [71]. Therefore, park planners and managers should pay more attention to the sign systems in urban parks to thereby meet people’s needs and to increase their satisfaction. 

Maintenances of facilities and plants in parks relate significantly to people’s satisfaction. This is consistent with previous studies that show that satisfaction not only depends on the availability of facilities and vegetation in urban parks, but also depends on their status in accordance with people’s various needs. The mere presence of facilities and vegetation is insufficient if there is bad maintenance [7]. It indicates that parks with good maintenance of facilities and plants usually have a higher degree of satisfaction. Good maintenance of facilities and plants in urban parks may provide security and encourage greater outdoor activities. 

Mosquito is another determinant factor that affects people’s satisfaction. Like sign system, mosquito is neither mentioned in previous studies nor in urban parks in other cities. Mosquitoes can spread malaria, dengue fever and many other diseases, which can cause serious threats to public health. The climate in Shenzhen, South China is warm with abundant rainfall, plentiful vegetation and longer mosquito activity periods; consequently, mosquitoes pose greater threats to residents’ health. The features of urban parks, such as water body, vegetation, and drainage systems, etc., can provide favorable conditions for mosquitos’ breeding and survival. Poor sanitation in some parks makes the situation even worse. Online comments show absolutely no positive emotions in regards to mosquitos in urban parks, and negative comments are assigned to a variety of comments including reports like “there are so many mosquitoes in the woods, suggesting bring a small tent and a mosquito-repellent spray” and “the zoo is smelly and full of mosquitoes in the middle of summer”. 

Urban parks provide relief from the crowd and polluted urban environment, and thus contribute to good physical and psychological health. Our studies show that the absence of or poor environmental cleanliness erode people’s satisfaction and discourage the use of parks. This finding supports previous studies [10]. For example, litter on trails may negatively influence the entire recreational experience [72]. Meanwhile, poor environmental cleanliness may be a signal of little management, which may include causal agents in evoking fear of crime [19]. Typical negative comments about environment cleanliness are like “trash can is hard to find, there is much litter on the ground”. It indicates that insufficient sanitation facilities, which impact the environmental cleanliness of urban parks, will then subsequently corrupt people’s satisfaction.

Urban parks provide critical ecological benefits to residents. They provide habitats, mitigate air pollution, reduce noise and alleviate urban heat-island effects [73,74,75,76]. Among the ecological benefits, air quality is an important factor that significantly affects people’s satisfaction with urban parks in Shenzhen. China’s tremendous economic growth in the past four decades has resulted in many environmental problems, especially the deterioration of air quality. Research has shown that air pollution exposure poses negative health impacts, including increased lung cancer incidence, cardiovascular morbidity and mortality [77]. There is growing evidence regarding the benefits of urban parks, especially air quality promotion [78]. Therefore, many residents come to parks to breathe fresh air, and this is probably the main reason why air quality significantly affects people’s satisfaction. Typical positive comments about air quality of urban parks is “when I have time, I will go to the park and walk around. A place with beautiful scenery and fresh air”. 

Vegetation is an important component; it significantly affects people’s satisfaction with urban parks in Shenzhen, which is consistent with previous studies in other cities [12,13]. Numerous studies demonstrate a wide range of ecological benefits that urban vegetation can have on the quality of life, including pollution mitigation, shade provision, and mitigation of heat islands. As China is one of the countries with the largest scale and accelerating speed of urbanization in the world, a series of ecological problems, especially air pollution, appear in many cities. It intensifies various demands on the vegetation of urban green spaces [74]. In Shenzhen, a coastal city in Southern China, the air quality in general is not bad and air pollution is not serious because of its geographical position. However, top air temperatures usually exceed 30 °C from May to October every year. High temperatures severely affect residents’ daily life and hinders people’s outdoor activities. All indoor places, in contrast, provide air conditioning for people. They become refuges for escaping threatening heat. However, it is not good for people to stay in this unventilated ambience every day. Vegetation in urban parks provides shade and mitigates solar heat gain, and thus is a well-documented ecological service with important implications for urban life. It leads to beneficial cooling effects and promotes outdoor activities [79]. Our observation is that people in Shenzhen want to be in green space, the presence of abundant plants and large tree canopies alleviates thermal discomfort. Besides, our findings may also reflect that preferred aesthetic environments for outdoor activities. Plant-filled spaces may be more appealing than those with different predominant landscape elements. 

Size is an essential character of urban parks. It also significantly affects people’s satisfaction in our study. Previous studies find that park size significantly relates to the number of activities in parks and determines the extent of ecosystem service provision. For instance, large parks generally have more recreation facilities, more plants, and larger water body, and thus people use larger parks more frequently, even if both larger and smaller parks are within a reasonable distance [34]. Small parks, such as community parks and neighborhood parks, may be important and suitable for daily recreation, frequent physical exercise or dog walkers in the early morning and late afternoon. However, their role in providing environmental benefits, such as mitigation of air pollution and heat islands, buffering noise from the surrounding urban environment, and filtering excessive rain after flush storms may have limits [80]. We found that larger parks generally have more online comments than small parks. Additionally, larger parks often have more organized activities that are likely to attract more people’ and therefore more online comments. For example, the music festival in Shenzhen Bay Park every summer, the chrysanthemum exhibition in Donghu Park every winter, the lotus flower show in Honghu Park every summer, the fireworks show in Shenzhen Bay Park in National Day every year, and the lighting show in Lianhuashan Park and Citizen Central Park every weekend. 

Landscape visual quality is considered to be an important feature of urban parks and is also an important part of the cityscape. Visual aesthetic quality is valuable for people’s physical and psychological health and is important for increasing the tourism potential of parks. Concurrent with previous studies that find good landscape visual quality can attract people, the study finds it significant in affecting people’s satisfaction with urban parks [81]. Landscape visual quality is unique and normally difficult to articulate, as it involves physical landscape and human perception of landscape. In some existing literature, the visual quality of physical landscape in parks means physical landscape elements, such as hills, water fountains and flowers. It also includes the facilities for culturally diverse activities, fields for sports activities, grass/lawn areas for sitting, hiking trails and retail shops. However, people’s perceptions about landscape would be changeable following the psychological paradigm, a process of interaction between the physical characteristics of landscape and the psychological responses of its users [82]. While landscape visual quality is important for people’s satisfaction with urban parks, ongoing maintenance that keeps landscapes in good condition is a significant component of landscape visual quality.

Recreational facility, such as fitness facility, sports field and amusement equipment, is the important element of comprehensive parks. Previous studies indicate that the recreational facility contributes to creating physically active urban lifestyles and physical health [83]. Concurrent with previous studies in which recreational facility has a positive relationship with the number of urban visitors, the study finds recreational facility significantly affecting people’s satisfaction with urban parks. We also find that parks, if charging admission fees for the use of recreational facilities, have more positive emotional responses than those providing recreational facilities for free. Parks charging fees for use presumably have more maintenance of facilities and more attractive recreational facilities, which is likely to attract more visitors and gain more acceptance. 

Several variables show no significant effect on people’s satisfaction with urban parks in Shenzhen. We find that two accessibility factors, traffic to parks and parking conditions, had no significant influence in this sense. Accessibility of urban parks, as is suggested in most studies, plays a positive role in attracting visitors [84]. Accessibility factors have no significant relationship to people’s satisfaction with urban parks in our study. The reason may be that some parks have good accessibility, so traffic to the park and parking conditions receive no special attention. Additionally, other parks with no convenient bus or subway service nor sufficient parking lots have few visitors, which causes fewer online comments on social media platforms. Three physical attributes (terrain, path and water body) and three facility factors (sanitation facility, rest facility and commercial facility) also have no significant influence on people’s satisfaction with urban parks. These results might imply that these factors in urban parks of Shenzhen are of good quality. Therefore, they are not of particular concern of visitors, and they are subsequently not popular in online comments. In other words, these factors are not important predictor variables of people’s satisfaction with urban parks in Shenzhen.

### 4.2. Discussion of Methodology and Future Research 

This paper uses online comment data from social media as a proxy to explore people’s satisfaction with urban parks, and uses several comprehensive parks of Shenzhen as case studies. One of the strengths of our study is that it addresses a gap in the existing literature, as suggested by numerous recent studies [7]. A further strength of our study is the innovative use of data extracted from social media as a potential proxy of people’s perception of and satisfaction with urban parks. Comparing with those measured by field surveys and interviews, to use Dianping data in a range of urban-related research appears applicable. In contrast to costly, time-consuming and labor-intensive methods as former, this new method is more time-efficient and can provide greater spatial and time coverage. This method also provides an unobtrusive method of non-participation, which is easy to replicate and thus seems promising for a future standardized approach. Being free, publicly available and instantly accessible, the data collection method using Dianping incurs no financial cost and takes significantly less time. The Dianping data without word limit mean that the analysis is in depth and more comprehensive than other social media such as Twitter with a limit of 140 characters. The attributes mentioned above claim that Dianping data are effective to provide information on the human–environment relationship in urban research.

It is clear that social media data has an enormous potential to understand people’s perception of and satisfaction with urban parks. However, this method also raises issues and limitations for future application. Firstly, due to little access to field surveys and interview data of urban parks, we are unable to test whether a good correlation exists between the comment data from social media and those data from actual field surveys and interviews. Secondly, the highly skewed nature of social media data, with a small number of urban parks contributing to the majority of online comments, raises concerns of representativeness. For example, visitors to famous and large parks, such as Lianhuashan Park and Shenzhen Bay park, might be more likely to Dianping in social media, but not likely to do so at “not so popular” and small parks. This potential difference among parks may contribute to the inaccuracy in the analysis of the effects of influential factors. The reliability and usefulness of Dianping data will be further validated if with large-scale field surveys publicly carried out by city authority in the future.

Additionally, the online comment data from social media limits the base population under investigation. There is a need to discuss the inherent biases in the dataset [39]. Dianping users may not be representative of all park users in the study area. Chinese internet-relevant research reports that an average age of Chinese “netizens” is 33, and 80% of Chinese “netizens” aged between 10 and 49 [85]. Crucially, the sample population does not include those urban populations that do not connect to the Internet. It is not consistent with the reality that parks are equally used by various sectors of the population, such as older people and children accounted for by the field survey. It also shows that the socio-demographic characteristics, such as age, gender, income and education level of Dianping users might differ from those of the whole population. Internet-relevant research also reports that China is the world’s largest Internet market and has 904 million internet users by March 2020. In terms of population structure, male and female netizens account for 51.9% and 48.1% respectively. The group with secondary education levels (junior middle school to junior college) and middle incomes (monthly income between 2001 and 5000 RMB) is the main force of the whole Internet users [85]. We can infer that at least age (both very old and very young population) and income (mostly poorer and richer populations) defies the even rates of social media use. Therefore, those social media users of Dianping may not be representative of park users as a whole. Such effects may potentially result in bias and may have to be taken into consideration. It is undoubtedly true when interpreting results from the use of such data as proxies for people’s perception of and satisfaction with urban parks. Continued investigation and discussion of bias are needed for park planning and management. Similar patterns found in this paper, if compared to previous studies, suggest that online comment data could be reliable to approximate people’s perception; however, it requires comparison and combination of multiple approaches, to see a broader picture of the relationship between people and urban environment.

The main limitation in our study, besides the general one of representativeness, is the low number of comments with sentiments and emotions. When we extract them from every sentence of each comment, most of the sentences are classified as neutral or of no identifiable emotion. It strongly affects the number of comments used for the subsequent analysis. Additionally, for online comments used as proxies for people’s perception and satisfaction in our study, the method of sentiment analysis has to deliver fast, accurate and replicable annotation and extract emotions from comments. The study adopts the method of manual annotation and extracting emotion. Manual annotation previously cited may provide the most reliable method of sentiment analysis, given that human annotators have the best chance of identifying the emotions present in comment texts [86]. However, a dataset resulting from manual annotation is not completely accurate, given that labelled comments with an emotion remain a subjective task [87]. Different human annotators may interpret the same text differently for many reasons. This issue is also relevant for the semi-supervised learning method used here, given that the “gold standard” tweet dataset used to train the algorithm relies on initial manual annotation of 1000 tweets [50]. A metric of comparison is derived to ensure the annotations are reliable between different human annotators. It suggests the agreement between them to be sufficient to assume inter-annotator reliability [69]. Kappa Indexes enable the assessment of inter-annotator reliability between different manual annotators and allow the variation in annotation by different annotators to be quantified. Besides inherent subjectivity, the most significant limitation of manual sentiment analysis of comments is time. Researchers need to examine every sentence of each comment. Given the large number of comments in our study, it takes a long period to complete manual annotation and extracting emotion. In further studies, automated and semi-automated methods may be better options. 

The regression model explains 85% of the total variation, indicating that additional factors not included in this study might also affect people’s satisfaction with urban parks. People’s satisfaction can be influenced by an individual’s socio-demographic (e.g., age and gender) and socio-economic characteristics (e.g., income, occupation and education level), as well as physical and psychological factors (e.g., disability, mental illness), as reviewed in the Introduction. Additionally, the majority of the online comments come from urban residents, yet still others from foreign tourists. The spatial variation of these user-based characteristics is a potential driving factor of satisfaction variance. Nevertheless, we found that the spatial quantification of these factors impose significant challenges at present. Unlike in field surveys and interviews, we are unable to obtain the sociodemographic and socioeconomic characteristics of Dianping users because of privacy protection, which hinders the further study of the relationship between people’s satisfaction and user-based factors.

## 5. Conclusions

This paper presents a method to investigate people’s perception of and satisfaction with urban parks by using geotagged online comments as a dataset. Dianping turns out to be a source of online comment data gathered through crowdsourcing. This study uses online comment data as a proxy for people’s perspective on urban parks, and Dianping data is useful in providing valuable information for exploring human-environment relationship in urban research. 

This study uses online comment data to identify a range of potential factors by textual and sentiment analysis that influences people’s satisfaction with urban parks. We found that, in addition to the common factors found in previous studies, such as accessibility, park size and recreation facility, a series of qualitative and contextual factors also influence people’s satisfaction. For instance, the sign system and mosquito rarely appear in previous studies about the human-urban park relationship. We found that nine predictor variables, including park size, air quality, vegetation, mosquito, recreation facility, sign system, landscape visual effect, maintenance of facilities and plants and environment cleanliness, significantly affect people’s satisfaction with urban parks. Among these, the sign system has the strongest influence, followed by the maintenance of facilities and plants, mosquito, environment cleanliness, air quality, vegetation, park size, landscape visual effect and recreation facility. Our findings increase the understanding of the human-urban park relationship and identify park characteristics that facilitate the degree of satisfaction promotion. 

Using online comment data from a social media platform to estimate people’s perspectives on urban parks herein offers several benefits. In comparison to traditional research methods, such as visitor surveys and interviews, this new method proves applicable. There is a need to identify several confines for more appropriate and effective use. It includes biases in the received datasets and a lack of sociodemographic and socioeconomic information about individuals included in the dataset.

## Figures and Tables

**Table 1:** The sample of information included in the comments data.

Site	User ID	Time	Score	Text	Photograph
Lianhuashan Park	A	5 June 2019 08:21	4	Here is the best park for picnics and kite flying. Large lawn and big trees, so it completely has not to worry that the shade place is not enough. And if having more time can also stroll Guan Shanyue art museum and Shenzhen Library at the foot of Lianhua mountain, and Shenzhen Museum and Shenzhen contemporary art and urban planning museum are in a little farther away. There is one of the best places to go.	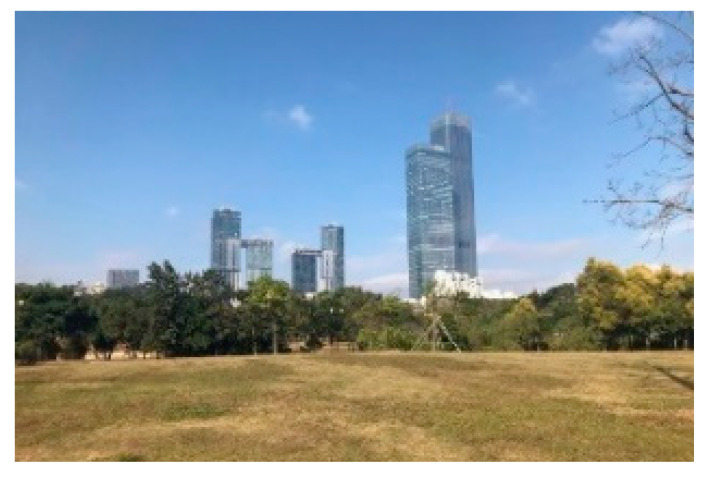 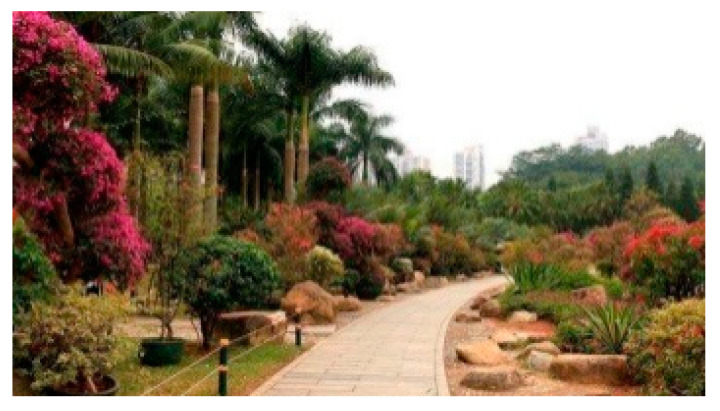 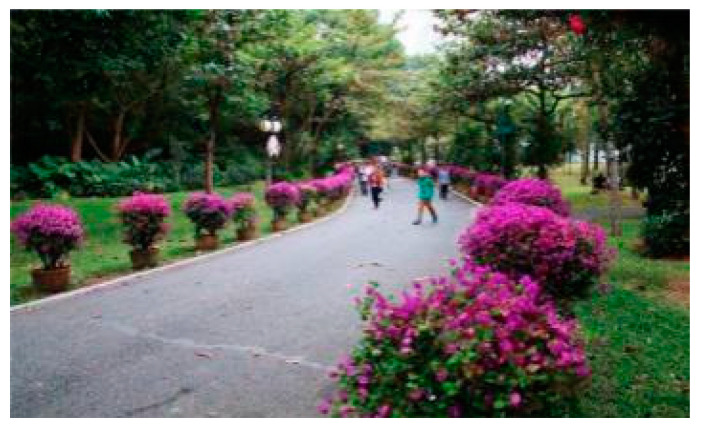

**Table 2:** The number of comments and the mean score of users’ satisfactions of each park with the number of comments exceeding 30.

Park	Number of Comments	Mean Score of Users’ Satisfactions	Park	Number of Comments	Mean Score of Users’ Satisfactions
Cuizhu Park	71	4.07	Sihai Park	157	4.07
Donghu Park	670	4.31	Moon Bay Park	46	4.15
Honghu Park	687	4.41	Zhongshan Park	226	4.54
People Park	293	4.29	Yantian Central Park	33	4.58
Bijia Hill Park	487	4.28	Baoan Park	125	4.37
Honggang Park	105	4.00	Xinan Park	35	3.77
Lizhi Park	682	4.37	Lingzhi Park	73	3.84
Lianhuashan Park	2244	4.51	Pingluanshan Park	44	4.48
Meilin Park	69	4.39	Tiezishan Park	38	3.95
Futian Ecology Park	146	4.38	Dayun Natural Park	31	4.35
Central Park	309	4.24	Henggang People Park	37	4.11
Citizen Central Park	550	4.65	Longcheng Park	65	4.45
Nanshan Park	335	4.38	Longcheng Square	107	4.26
Dashahe Park	173	4.32	Long Park	64	4.03
Lilin Park	49	4.27	Shiyaling Park	34	3.88
Lixiang Park	188	4.31	Longhua Park	98	3.95
Qianhaishi Park	34	4.38	Julongshan Park	54	4.19
Shenzhen Bay Park	2511	4.55	Honghuashan Park	73	4.19

**Table 3:** The results from multiple linear regressions of people’s satisfaction with parks on predictors.

Variable	Coefficient	Standardized Coefficient	Significance	VIF
(Constant)	3.986	0.083	0.000	
Park size	0.412	0.142	0.007	1.438
Air quality	0.841	0.232	0.001	2.067
Vegetation	−1.018	0.204	0.000	1.982
Mosquito	2.386	0.860	0.010	1.542
Recreational facility	0.330	0.118	0.010	1.438
Sign system	5.049	1.304	0.001	1.747
Landscape visual quality	0.666	0.124	0.000	2.549
Maintenance of facilities and plants	1.758	0.948	0.037	1.813
Environment cleanliness	1.767	0.585	0.006	1.342
*R* ^2^	0.850	-	-	-
Adjusted *R*^2^	0.798	-	-	-
Mean VIF	-	-	-	1.769

## Data Availability

3rd Party Data.
